# A Prospective Multicenter Evaluation of the Accuracy and Safety of an Implanted Continuous Glucose Sensor: The PRECISION Study

**DOI:** 10.1089/dia.2019.0020

**Published:** 2019-05-07

**Authors:** Mark P. Christiansen, Leslie J. Klaff, Timothy S. Bailey, Ronald Brazg, Grace Carlson, Katherine S. Tweden

**Affiliations:** ^1^ Diablo Clinical Research, Walnut Creek, California.; ^2^ Rainier Clinical Research Center, Inc., Renton, Washington.; ^3^ AMCR Institute, Inc., Escondido, California.; ^4^ Carlson Consulting, San Francisco, California.; ^5^ Senseonics, Inc., Germantown, Maryland.

**Keywords:** Type 1 diabetes, Type 2 diabetes, Continuous glucose monitoring, Implantable, accuracy

## Abstract

***Background:*** A prior study (PRECISE II) demonstrated that an implantable continuous glucose monitoring (CGM) system (Eversense^®^ CGM System) provided accurate glucose readings through the 90-day sensor life with a favorable safety profile in participants with type 1 or type 2 diabetes (T1D, T2D). This study was performed to further characterize the accuracy of the system.

***Methods:*** PRECISION was a prospective multicenter study that evaluated the accuracy and safety of Eversense among adults with T1D or T2D through 90 days (NCT02647905). Accuracy measures included percentage system agreement and mean absolute relative difference (MARD) between Eversense and Yellow Springs Instrument reference measurements from 40 to 400 mg/dL. The primary safety endpoint was incidence of device-related or sensor insertion/removal procedure-related serious adverse events (SAEs) through 90 days. An updated glucose calculation algorithm was also applied to the sensor data from the PRECISE II study to evaluate consistency of accuracy results.

***Results:*** Thirty-five participants received the CGM system. Eighty-five percent of CGM values were within 15/15% of reference and the MARD value against reference was 9.6% (95% confidence interval [CI]: 8.9–10.4). All sensors were functional through day 90. No device- or procedure-related SAEs occurred. Application of the updated algorithm to PRECISE II sensor data resulted in 87% of readings within 15/15% of reference and an MARD value against reference of 8.5% (95% CI: 8.0%–9.1%).

***Conclusions:*** PRECISION corroborated prior accuracy and safety findings of the Eversense CGM System through the 90-day sensor life. The updated algorithm improved accuracy of measurements in PRECISE II.

## Introduction

Real-time continuous glucose monitoring (CGM) has been shown to be superior in improving glycated hemoglobin (HbA1c) and reducing time spent in hypoglycemia compared with usual care with home blood glucose (BG) meters in individuals with type 1 diabetes (T1D)^1–3^ and type 2 diabetes (T2D).^4,5^ Prior studies have demonstrated that consistent use of CGM is required to effectively lower HbA1c and time in hypoglycemia; this improvement was negated when CGM was discontinued.^1,2^ Despite the known benefits of consistent use of CGM, a recent survey of individuals who initiated CGM in the T1D Exchange registry found that 27% of patients discontinued use during the first year.^6^ Commonly reported reasons for discontinuing the use of transcutaneous CGM systems include problems with the CGM functionality or providing inaccurate information (71%), problems with the sensor insertions or adhesion (61%), lack of insurance coverage (58%), discomfort wearing the sensor (41%), already using a pump and not wanting to use another invasive device (33%), and the large size of the CGM device (28%).^6^

An implantable subcutaneous CGM system (Eversense^®^ CGM System; Senseonics, Inc., Germantown, MD) was developed to address several of the limitations of use of conventional CGM systems. The Eversense sensor is designed to be inserted in the subcutaneous tissue of the upper arm and to function for up to 90 days, which is intended to reduce the inconvenience and discomfort of weekly sensor insertions and to provide long-term wear convenience. The Eversense smart transmitter is worn over the sensor to wirelessly power it to measure and calculate glucose and to directly transfer the data to a Mobile Medical Application (app) on a smartphone. The transmitter, which is kept in place with a mild silicone-based adhesive that is changed daily, can be removed at any time without the need for sensor replacement. When user-defined hyperglycemic and hypoglycemic glucose thresholds are reached, the system provides audio and visual alerts and notifications by the app and on-body vibratory alerts from the transmitter that are independent of the app.

The first version of the Eversense CGM System was evaluated in a multicenter European 180-day pivotal study (PRECISE), which demonstrated that use of the CGM device reduced mean glucose and HbA1c levels compared with baseline in 71 participants with T1D or T2D.^7^ The PRECISE study also established the accuracy against reference venous glucose values in the range of 40–400 mg/dL with a mean absolute relative difference (MARD) of 11.6%.^7^ The data from the PRECISE trial were used as a training set to further improve the glucose calculation algorithm used within the system. Subsequently, the multicenter U.S. pivotal PRECISE II trial evaluated the accuracy and safety of an updated Eversense CGM System, including the modified glucose calculation algorithm and a new sensor configuration, in 90 participants with T1D and T2D for up to 90 days. The overall MARD value against reference glucose values was 8.8%.^8^ Eighty-six percent of CGM values were within 15/15% of reference values over the total glucose range of 40–400 mg/dL.^8^ After the conduct of the PRECISE II study, the algorithm was further refined using the data from the PRECISE study to improve accuracy particularly in the early sensor life and hypoglycemic range.

In this report, findings are presented from the prospective multicenter nonrandomized unblinded PRECISION study, which further evaluated the accuracy and safety of the Eversense CGM System for up to 90 days with emphasis on performance in early sensor life and in the hypoglycemic range in individuals with T1D and T2D. To provide additional evidence for validation, the updated algorithm was also applied in a post hoc manner to the raw sensor data collected in the PRECISE II study to evaluate differences in the accuracy measures compared with the prior algorithm.

## Methods

### Study design and participant enrollment

PRECISION was a prospective unblinded nonrandomized multicenter study including adult participants with T1D and T2D to evaluate the safety and accuracy of the Eversense CGM System up to 90 days. The study was conducted between July 2017 and February 2018 at three sites in the United States. Individuals were eligible for participation in the study if they were at least 18 years of age and had a clinically confirmed diagnosis of T1D or T2D for at least 1 year. Individuals were excluded from participation if they had any of the following: a history of severe hypoglycemia or diabetic ketoacidosis, necessitating an emergency room visit or hospitalization during the previous 6 months; a condition complicating sensor placement, operation, or removal; symptomatic coronary artery disease, unstable angina, myocardial infarction, or stroke in the previous 6 months; uncontrolled hypertension; hematocrit <30% or >50%; lactation or pregnancy during the study; presence of other active implanted devices; or a condition likely to require magnetic resonance imaging (MRI) for the duration of the study.

The study was performed in accordance with the Declaration of Helsinki and was approved by a centralized internal review board. All participants provided both verbal and written informed consent.

### Study device

The Eversense CGM System consists of an implantable fluorescence-based cylindrical glucose sensor (3.5 × 18.3 mm), a smart transmitter, and MMA (app) that displays glucose information in real time and operates on a mobile device.

The sensor technology has been described in detail previously.^8^ In brief, it contains core electronics and optics that are sealed within a poly(methyl methacrylate) encasement. The sensor is activated to measure interstitial fluid glucose every 5 min when it receives radiofrequency power from the transmitter. A fluorescent hydrogel-based copolymer matrix is grafted to the outside of the encasement, which is designed to reversibly bind glucose to detect changes in glucose concentrations. Glucose binding results in an increase in fluorescence intensity, which is measured by the sensor's optical system. The fluorescence data are sent to the transmitter, where glucose is calculated, and the integrity of the system is checked. The sensor has a silicone collar impregnated with ∼1.75 mg of dexamethasone acetate, which elutes an average of 3 μg per day over the life of the sensor,^9^ to diminish the body's local inflammatory response to prolong the sensor life. A sensor replacement alert is provided to the user when the system reaches inadequate glucose sensitivity due to oxidative degradation of the glucose binding chemistry to safeguard the system accuracy throughout sensor life.^10^

The transmitter (37.6 by 48.0 mm, 8.8 mm thick, 11.3 g), which contains a battery as the only power source, is placed on top of the skin over the sensor and transfers glucose data to the app every 5 min through a secured low-energy Bluetooth transmission. The transmitter, which requires recharging approximately every other day for 15 min, is customizable to vibrate distinctively in response to a variety of conditions (hypoglycemia, rapid rate of change, etc.) to alert the user. It is water resistant to a depth of one meter submerged for 30 min.

The app displays glucose values, trends, and alerts information to the user. The user can also set customized glucose target ranges and alert levels (including predictive and rate-of-change alerts). The glucose calculation algorithm was updated following the PRECISE II study^8^ to improve accuracy in early sensor life and in the hypoglycemic ranges.

### Study procedures

The study consisted of the following visits: baseline screening; sensor insertion (day 0); six accuracy assessments at days 1, 7, 14, 30, 60, and 90; and a postsensor removal follow-up assessment (up to 10 days after removal). A subgroup of subjects had two sensors inserted, one in each arm.

At the baseline screening visit, investigators obtained participant demographics and medical history and performed laboratory measurements (i.e., hemoglobin A1c, hematocrit, and plasma dexamethasone), a physical examination, and an electrocardiogram. Urine pregnancy testing was also conducted in female participants. Sensors were inserted into the upper arm at the sensor insertion visit (day 0) by trained providers (i.e., physicians, nurse practitioners, or physician assistants). At all visits after baseline screening, investigators assessed adverse events (AEs), sensor insertion sites, hematocrit levels, pregnancy status, and changes in medications.

Details of the sensor insertion procedure have been previously described.^8^ In brief, using sterile technique, the study team disinfected and locally anesthetized the incision and pocket site and then made a 5 mm incision ∼3 to 5 mm deep. Next, a subcutaneous pocket ∼3 to 5 mm deep and greater than the length of the sensor was made from the incision toward the shoulder using a custom-designed blunt tissue dissector. The sensor was placed into the pocket using an insertion tool designed to protect the sensor during deployment. The incision was closed using Steri-Strips™.

The participant was prompted to begin calibration 24 h after insertion. Transmitter(s) were worn over the sensor(s) and participants were prompted by the app to perform calibrations twice a day using the Accu-Chek^®^ Aviva Plus BG meter and respective test strips (Roche Diabetes Care, Inc., 8900 Hague Rd, Indianapolis, IN). Participants were advised to perform seven fingersticks a day. The BG meter data were downloaded at each follow-up visit. Participants and investigators were able to see CGM values including all alerts and prompts from the app for the duration of the study; however, all diabetes care decisions were based on current clinical standards of care using BG meter data.

Accuracy was evaluated by comparing the CGM system glucose values with those of venous blood samples measured by a reference glucose analyzer (2300 Stat Plus Glucose and Lactate Analyzer; Yellow Springs Instruments [YSI], Yellow Springs, OH). Participants on insulin and without gastroparesis underwent hypoglycemia and hyperglycemia challenges during accuracy assessment visits wherein participant's glucose levels were raised, using mixed meals of 30%–40% carbohydrate content, or lowered, using subcutaneous insulin dosing based on each participant's individualized insulin sensitivity, for prescribed periods of time to evaluate the sensor performance over the range of 40 to 400 mg/dL. Venous reference blood samples were drawn for a 16-h period on days 1, 7, and 14 and for a 12.5 h period on days 30, 60, and 90. Based on the participant's BG level, samples were drawn every 5–15 min (every 15 min for BG >75 mg/dL and <325 mg/dL, every 5 min for BG ≤75 mg/dL and ≥325 mg/dL). Each reference glucose value was paired to the corresponding CGM glucose obtained within 5 min after the blood draw. Participants maintained their diabetes treatment routine throughout the visits.

Participants with one sensor inserted into the left arm had blood samples drawn at 30 min, 2, and 4 h postinsertion and then daily for the first 8 days of sensor wear for additional plasma dexamethasone evaluation. Participants who had two sensors inserted, one in each arm, underwent blood draws for dexamethasone evaluation 2 h postinsertion. All participants had blood samples drawn for dexamethasone assessment at each visit and during each calendar day at the visits that spanned 2 days (i.e., days 1, 7, and 14). The assay used to measure dexamethasone in plasma had a detection limit of 0.05 ng/mL. During the screening visit and at 30, 60, and 90 days, venous blood samples were obtained for HbA1c levels.

The sensors were removed after the 90-day visit by repeating a small 5 mm skin incision and retrieving the subcutaneous sensor. Participants returned ∼10 days after removal for follow-up to assess the healing of the removal site.

### Study outcomes

The accuracy measures included CGM system agreement within specific percentages of the reference glucose values, MARD for paired sensor and reference glucose measurements, accuracy by study visit, and alert performance collected during the clinic visits through 90 days postinsertion across the glucose range of 40–400 mg/dL. The CGM Satisfaction Scale Questionnaire was administered at the end of the study to assess participant ratings of device accuracy, wearability, functionality, and overall satisfaction on a 5-point Likert scale with a greater value denoting higher satisfaction or less hassle.^11^ Sensor longevity and transmitter wear time were also evaluated.

The safety endpoint was the rate of device-related or sensor insertion/removal procedure-related serious AEs (SAEs) throughout the study, including sensor removal and final follow-up visit. Other safety objectives were to evaluate the incidence of all procedure-related or device-related AEs, and all AEs regardless of relatedness during in-clinic sessions and home use. All reported AEs were adjudicated by an independent medical monitor for relatedness to the device, sensor insertion/removal procedure, and study procedure (e.g., hyperglycemia and hypoglycemia challenges). AE severity was graded by the site principal investigator.

### PRECISE II study analysis

The updated glucose calculation algorithm was applied to the raw sensor data obtained during the PRECISE II study (*n* = 90 participants).^8^ The accuracy measures of MARD and CGM system agreement within specific percentages of the reference glucose values were calculated.

### Statistical methods

The prespecified analysis population for the effectiveness measures was based on all evaluable glucose data from all participants with at least one paired glucose reading. The safety analysis population included all participants who had a sensor placed.

All analyses were evaluated using descriptive statistics and 95% confidence intervals (CIs); no formal hypothesis testing was conducted.

## Results

Thirty-six participants were enrolled and 35 participants (*n* = 8 single sensor and *n* = 27 bilateral sensors) were inserted with the sensor and are included in the effectiveness and safety populations. One participant withdrew from the study after enrollment and before insertion. Sixty-two sensors were placed in the study (8 single sensor participants, 27 bilateral dual sensor participants). All 35 participants who had a sensor inserted completed the study. Participant baseline characteristics are presented in Table 1.

**Table 1.** Baseline Participant Characteristics (*n* = 35)

*Variable*	*Efficacy/safety population*
Gender, *n* (%)	
Male	18 (51.4)
Female	17 (48.6)
Age, years (SD)	51.6 (15.7)
Ethnicity, *n* (%)	
Hispanic	4 (11.4)
Non-Hispanic	31 (88.6)
Race, *n* (%)	
Caucasian	32 (91.4)
Black or African American	1 (2.9)
Asian	2 (5.7)
BMI, kg/m^2^ (SD)	28.2 (5.4)
Years since diabetes diagnosis, years (SD)	26.0 (14.3)
Diabetes type, *n* (%)	
Type 1	25 (71.4)
Type 2	10 (28.6)
Type of diabetes therapy, *n* (%)	
Oral or diet and exercise^a^	5 (14.3)
Multiple daily insulin injections	11 (31.4)
Continuous insulin infusion pump	19 (54.3)

^a^ Participants with T2D.

SD, standard deviation; T2D, type 2 diabetes.

The average time for the sensor insertion procedure was 2.3 min and the average time for the sensor removal procedure was 4.5 min. All sensors were functional through day 90 for a total of 6148 days of sensor exposure. The median wear time was 23.4 h per day over the full 90-day period, or 98% of the time. Hemoglobin A1c levels at baseline were 7.8% (standard deviation [SD] = 1.3) and 7.5% (SD = 0.9) at 90 days for a mean change of 0.3 percentage points (SD = 1.0).

### Accuracy

A total of 15,170 matched glucose pairs were collected. The MARD over the glucose range of 40–400 mg/dL was 9.6% (95% CI: 8.9%–10.4%). Seventy-nine percent of CGM values were within 15/15% of reference on day 1; 84% to 88% of CGM values were within 15/15% at subsequent time points through end of sensor life (Table 2). The absolute deviation for each individual observation as a function of the YSI comparator glucose level and the Bland–Altman plots are shown in Supplementary Figures S1 and S2 to further characterize the accuracy and precision of the CGM system.

**Table 2.** Continuous Glucose Monitoring System Accuracy and Stability by Clinic Visit

*Day postinsertion*	*No. of paired CGM system-YSI reference readings*	*Percent within 15/15% reference*	*Percent within 20/20% reference*	*Percent within 30/30% reference*	*Percent within 40/40% reference*	*Accuracy MARD, %*
Day 1	2665	79	89	96	99	11.6
Day 7	2926	86	93	98	99	9.8
Day 14	2997	88	95	99	100	9.0
Day 30	2284	88	94	99	100	8.9
Day 60	2133	87	94	99	100	8.7
Day 90	2165	84	92	99	99	9.7

CGM, continuous glucose monitoring; MARD, mean absolute relative difference; YSI, Yellow Springs Instrument.

The percentage agreement using the 15/15% criteria was 81% or greater for all subsets of the glucose range and 85% overall (Table 3). Of particular note for the hypoglycemic range, CGM system agreement within 40 to 60 mg/dL and 61 to 80 mg/dL was 92% and 87% with corresponding MAD of 7.2 mg/dL and 7.6 mg/dL, respectively. In addition, the CGM system demonstrated accuracy across different rates of change (Supplementary Table S1).

**Table 3.** Continuous Glucose Monitoring System Accuracy Over Continuous Glucose Monitoring System Glucose Range

*CGM system glucose range (mg/dL)*	*No. of paired CGM system-YSI reference readings*	*Percent within 15/15% reference*	*Percent within 20/20% reference*	*Percent within 30/30% reference*	*Percent within 40/40% reference*	*Accuracy MARD %*^a^
Overall	15,170	85	93	98	99	9.6
40–60	1236	92	96	98	99	7.2
61–80	2003	87	94	99	100	7.6
81–180	5786	81	90	97	99	10.5
181–300	3566	85	93	98	99	8.6
301–350	1628	93	98	99	100	6.9
351–400	951	92	96	99	100	6.4

^a^ Mean absolute difference (mg/dL) was calculated for glucose values ≤80 mg/dL.

The system alert performance is shown in Table 4. Confirmed event detection rates at the threshold reference values of 70 and 180 mg/dL were 95% and 99%, respectively. Conversely, false alert rates of the CGM system at the same high and low threshold values were 8% and 7%, respectively.

**Table 4.** In-Clinic Hypoglycemic and Hyperglycemic Event Detection Using Both Threshold and 10 min Predictive Alerts

*Glucose setting (mg/dL)*	*Confirmed event detection rate*	*Missed event detection rate*	*True alert rate*	*False alert rate*
Hypoglycemic alert				
60	89%	11%	77%	23%
70	95%	5%	92%	8%
Hyperglycemic alert				
180	99%	1%	93%	7%
240	99%	1%	94%	6%

### Participant satisfaction

The mean overall satisfaction score on the inventory was 3.9 (SD = 0.5). The mean scores were 3.9 (SD = 0.6) on the benefit subscale and 4.1 (SD = 0.6) on the hassle subscale (the higher the scores, the more the benefit and less the hassle of the CGM system). Participants were allowed to provide free-form responses about the device. The most commonly reported positive attributes of the device included the convenience accessing BG reports, the help in controlling diabetes, having alerts for low BG levels, and freedom from having to replace the CGM weekly. The most frequently cited negative attributes of the device were the location and larger size of the first-generation transmitter, cases when the BG meter and CGM reported different glucose values, charging the transmitter, and the frequency of alarms. Overall, only 20% of participants reported being unwilling to use the device after the study was over.

### Safety

During the study, there were a total of eight AEs among five participants that were adjudicated as either related or possibly related to the device or insertion/removal procedures. Most events were dermatologic in nature, described as mild in severity and resolved without intervention. There were two events each of sensor location pain/discomfort due to the insertion or removal procedure (one mild and one moderate severity), skin discoloration (both mild severity), dermatitis (both mild severity), and difficulty removing the sensor (both mild severity). There were no incisional infections from the insertion or removal procedures and no device- or insertion/removal procedure-related SAEs.

There was a total of three AEs among three participants that were adjudicated as possibly related to the procedure (i.e., conduct of the hypoglycemic and hyperglycemic challenges at accuracy assessment visits). All three events were reported as headache secondary to low blood sugar and mild in severity.

As an additional safety measure, plasma dexamethasone levels were evaluated throughout the study. Plasma dexamethasone levels were undetectable (<0.05 ng/mL) for all participants before insertion. No subject with a single sensor had a plasma sample containing detectable levels of dexamethasone (detection limit of 0.05 ng/mL) at any time during the study. There were 18 of 27 (66%) subjects with two sensors that had detectable levels >0.05 ng/mL in the first 8 days after insertion. The maximum level detected was 0.114 ng/mL at day 2 in one subject that fell below the detection limit by day 7, that is, lower than the level of 1 ng/mL, which has been shown to produce a therapeutic effect.^11,12^ Through the 90-day study, no dexamethasone was detected in any subject after day 8.

### Updated glucose calculation algorithm for PRECISE II

The original MARD reported in PRECISE II using the prior glucose algorithm based on 15,753 matched CGM and reference pairs was 8.8% (95% CI: 8.1%–9.3%).^8^ The CGM system agreement to reference within YSI glucose ranges is given in Table 5. The updated algorithm, when applied to the raw primary sensory data in PRECISE II, resulted in an MARD of 8.5% (95% CI: 8.0%–9.1%). Eighty-seven percent of CGM system readings were within 15/15% of reference values across 40–400 mg/dL. A performance improvement from 83% to 89% was observed in the very low hypoglycemic range (<54 mg/dL) and a slight improvement from 86% to 87% was observed between the software versions at >54 to 71 mg/dL range (Supplementary Table S2).

**Table 5.** Continuous Glucose Monitoring System Accuracy Over Continuous Glucose Monitoring System Glucose Range of PRECISE II

*CGM system glucose range (mg/dL)*	*No. of paired CGM system-YSI reference readings*	*Percent within 15/15% reference*	*Percent within 20/20% reference*	*Percent within 30/30% reference*	*Percent within 40/40% reference*	*Accuracy MARD, %*
Overall	15,753	87	94	99	100	8.5
40–60	480	85	92	98	100	8.3
61–80	1111	83	91	97	99	8.7
81–180	7844	86	94	98	100	8.4
181–300	5377	88	96	99	100	7.8
301–350	692	91	98	100	100	7.0
351–400	249	97	99	100	100	5.2

## Discussion

The primary objectives of the PRECISION study were to evaluate the accuracy of the Eversense CGM System in measuring glucose early during the 90-day sensor life as well as in the hypoglycemic ranges. The system was shown to be accurate overall with an MARD of 9.6% over the glucose range of 40–400 mg/dL and 85% of CGM values within 15/15% of reference values. The first day after insertion, 79% of CGM values were within 15/15% of reference values and improved to 86% by day 7. Accuracy was demonstrated in the hypoglycemic range with 92% of readings within 15/15% of reference values in the glucose range of 40 to 60 mg/dL.

The favorable safety profile observed in PRECISE II^8^ was corroborated in this study. The insertion, use, and removal of 62 sensors in 35 participants resulted in a low burden of related AEs that were nearly all mild in severity. In addition, there were no detectable levels of dexamethasone in plasma of participants with one sensor, which is how the system will be used in clinical practice. Among participants who wore two sensors for the purposes of this study, the maximum level of dexamethasone was 0.114 ng/mL at day 2 postinsertion, which fell below the detection limit by day 7. This level is eight times lower than the 1 ng/mL level that is required to have a therapeutic effect,^12,13^ providing evidence that the risk of chronic dexamethasone exposure with the Eversense sensor is low.^9^

Participants wore the CGM system for a median 98% of the time supporting that Eversense, with a long-term implantable sensor, can fit within patients’ lifestyles. The high use of the device was consistent with findings on the CGM Satisfaction Scale, where four in five participants reported being willing to continue using the device after the conclusion of the study. Randomized controlled trials employing a cross-over study design have demonstrated that consistent CGM use resulted in improvement in HbA1c and time in range including a reduction in time spent in hypoglycemia.^1,2^ The results from this study also showed improvement in HbA1c consistent with these prior trials.

The results of the PRECISION study compare favorably with results from the PRECISE II study^8^ with 85% and 86% of readings within 15/15% reference glucose value overall, respectively, median transmitter wear times of 23.4 h per day in both studies, and high percentage of sensors lasting 90 days with 91% and 100% survival in PRECISION and PRECISE II, respectively. The safety profile was also similar with 14 device- or insertion/removal procedure-related events (in 7 of 90 participants) in PRECISE II^8^ and 8 device- or insertion/removal procedure-related AEs (in 5 of 35 participants) in PRECISION of the same general nature.

Application of the updated glucose calculation algorithm to the PRECISE II raw sensor data was consistent with the findings in PRECISION. The updated algorithm resulted in numerical improvement in the MARD from 8.8% to 8.5% and in the performance in the very low hypoglycemic range (from 83% to 89% at <54 mg/dL).

It is noted that people with diabetes would require serial sensor insertions and removals over the span of their life with any CGM system including Eversense. With Eversense, a small incision (∼5 mm long) is required to insert and remove the sensor. The insertion tools have been designed to minimize tissue disruption. Insertion and removal procedure-related AEs were mostly mild and resolved quickly. Clinical studies in the United States to date have evaluated only one cycle of insertion and removal; however, future studies will involve evaluating the accuracy and safety of serial insertions and removals for 2 years to demonstrate that the safety remains positive.

## Conclusions

The PRECISION study confirmed the accuracy and safety of the implantable continuous glucose sensor throughout the 90-day sensor life with additional accuracy data during early sensor life and in the hypoglycemic range. Study participants had high rates of sensor use throughout the sensor life.

## Supplementary Material

Supplemental data

Supplemental data

Supplemental data

Supplemental data
