# Supplementary material for: A Prospective Multicenter Evaluation of the Accuracy and Safety of an Implanted Continuous Glucose Sensor: The PRECISION Study
Source: Diabetes Technol Ther. 2019 May 7;21(5):231–7. doi: 10.1089/dia.2019.0020 (PMC6532543; doi:10.1089/dia.2019.0020)
Supplement: Supplemental data [file Supp_Fig1.pdf]

Supplementary Data

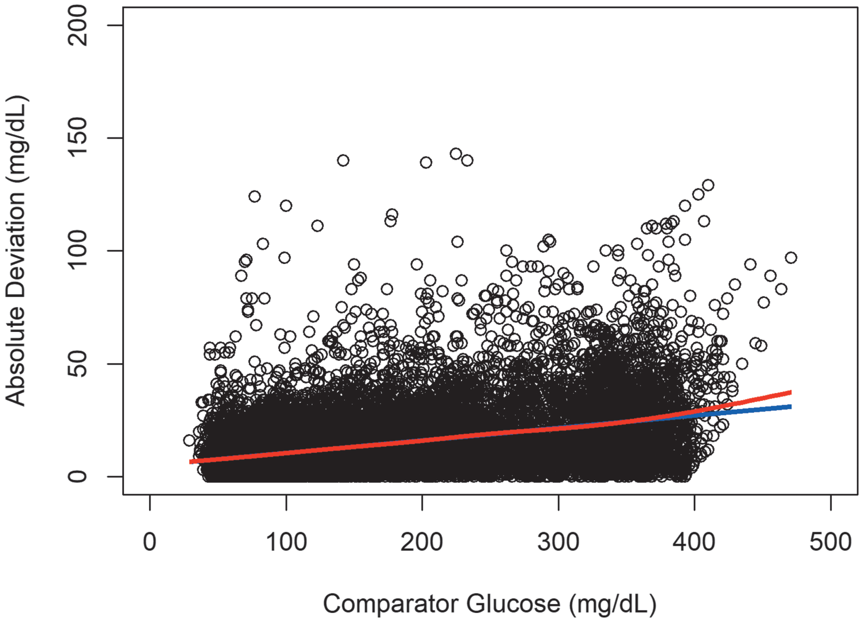

SUPPLEMENTARY FIG. S1. Absolute deviation between CGM and reference glucose with respect to reference comparator glucose. Linear best fit line in blue and nonlinear best fit line in red. CGM, continuous glucose monitoring.
