# Supplementary figures and images for: A Prospective Multicenter Evaluation of the Accuracy and Safety of an Implanted Continuous Glucose Sensor: The PRECISION Study
Source: Diabetes Technol Ther. 2019 May 7;21(5):231–7. doi: 10.1089/dia.2019.0020 (PMC6532543; doi:10.1089/dia.2019.0020)

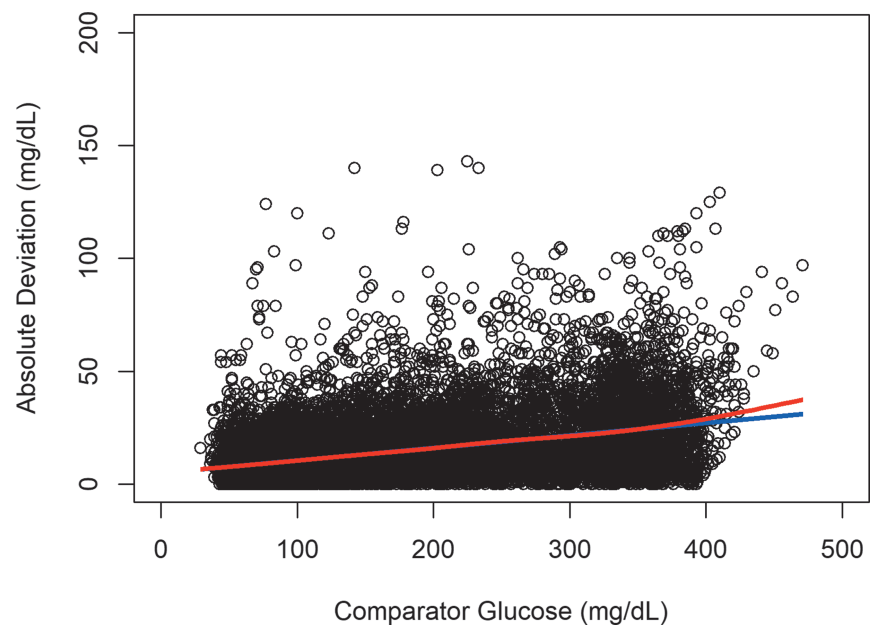

**SUPPLEMENTARY FIG. S2.** Bland–Altman plot.

Supplement: Supplemental data [file Supp_Fig2.pdf]
