# Supplementary material for: A Prospective Multicenter Evaluation of the Accuracy and Safety of an Implanted Continuous Glucose Sensor: The PRECISION Study
Source: Diabetes Technol Ther. 2019 May 7;21(5):231–7. doi: 10.1089/dia.2019.0020 (PMC6532543; doi:10.1089/dia.2019.0020)
Supplement: Supplemental data [file Supp_Table1.pdf]

Supplementary Table S1. PRECISION Study: Effect of System Rate of Change on CGM System and Reference Agreement

CGM system rate of change (mg/[dL·min])	*No. of paired CGM system-YSI reference readings*	*Percent of CGM system readings within*			
		15/15% of reference	*20/20% of reference*	*30/30% of reference*	*40/40% of reference*
Less than −2	475	79	91	97	99
(−2, −1)	1137	84	92	98	99
(−1, 1)	10,928	87	94	98	100
(1, 2)	1013	82	91	98	99
More than 2	539	76	87	97	99
