# Supplementary material for: A Prospective Multicenter Evaluation of the Accuracy and Safety of an Implanted Continuous Glucose Sensor: The PRECISION Study
Source: Diabetes Technol Ther. 2019 May 7;21(5):231–7. doi: 10.1089/dia.2019.0020 (PMC6532543; doi:10.1089/dia.2019.0020)
Supplement: Supplemental data [file Supp_Table2.pdf]

Supplementary Table S2. PRECISE II with Updated Glucose Calculation Algorithm Agreement to Reference

Reference glucose range (mg/dL)	*No. of paired CGM system-YSI reference readings*	*Percent of CGM system readings within*			
		15/15% of reference	*20/20% of reference*	*30/30% of reference*	*40/40% of reference*
≤54	224	89	94	99	100
>54 to <71	848	87	95	98	99
71 to 180	8122	85	93	98	99
>180	8122	85	93	98	99
Total	15,753	87	94	99	100
